# Comparing five equations to calculate estimated glomerular filtration rate to predict acute kidney injury following total joint arthroplasty

**DOI:** 10.1186/s42836-022-00161-4

**Published:** 2023-03-10

**Authors:** Kevin L. Mekkawy, Yash P. Chaudhry, Sandesh S. Rao, Micheal Raad, Raj M. Amin, Harpal S. Khanuja

**Affiliations:** 1 Department of Orthopaedic Surgery, The Johns Hopkins University School of Medicine, Baltimore, MD 21224 USA; 2 Department of Orthopaedic Surgery, Philadelphia College of Osteopathic Medicine, Philadelphia, PA 19131 USA; 3 Department of Orthopaedic Surgery, Stanford University Medical Center, Palo Alto, CA 94063 USA

**Keywords:** Total joint arthroplasty, Estimated glomerular filtration rate, Ecute kidney injury

## Abstract

**Background:**

Acute kidney injury (AKI) following total joint arthroplasty (TJA) is associated with increased morbidity and mortality. Estimated glomerular filtration rate (eGFR) is used as an indicator of renal function. The purpose of this study was (1) to assess each of the five equations that are used in calculating eGFR, and (2) to evaluate which equation may best predict AKI in patients following TJA.

**Methods:**

The National Surgical Quality Improvement Program (NSQIP) was queried for all 497,261 cases of TJA performed from 2012 to 2019 with complete data. The Modification of Diet in Renal Disease (MDRD) II, re-expressed MDRD II, Cockcroft-Gault, Mayo quadratic, and Chronic Kidney Disease Epidemiology Collaboration equations were used to calculate preoperative eGFR. Two cohorts were created based on the development of postoperative AKI and were compared based on demographic and preoperative factors. Multivariate regression analysis was used to assess for independent associations between preoperative eGFR and postoperative renal failure for each equation. The Akaike information criterion (AIC) was used to evaluate predictive ability of the five equations.

**Results:**

Seven hundred seventy-seven (0.16%) patients experienced AKI after TJA. The Cockcroft-Gault equation yielded the highest mean eGFR (98.6 ± 32.7), while the Re-expressed MDRD II equation yielded the lowest mean eGFR (75.1 ± 28.8). Multivariate regression analysis demonstrated that a decrease in preoperative eGFR was independently associated with an increased risk of developing postoperative AKI in all five equations. The AIC was the lowest in the Mayo equation.

**Conclusions:**

Preoperative decrease in eGFR was independently associated with increased risk of postoperative AKI in all five equations. The Mayo equation was most predictive of the development of postoperative AKI following TJA. The mayo equation best identified patients with the highest risk of postoperative AKI, which may help providers make decisions on perioperative management in these patients.

## Background

There has been a growing emphasis on preoperative medical optimization in total joint arthroplasty (TJA) patients to improve care and decrease the risk of postoperative complications. It is important to identify renal dysfunction perioperatively to properly stratify and modify care to improve outcomes. There are mixed reports on the incidence of acute kidney injury (AKI) after joint arthroplasty, ranging anywhere from 2% to 15% [1–3]. AKI following surgery has been shown to increase length of stay and costs related to complications, as well as increased mortality rates [4, 5]. One of the most widely accepted classifications, Kidney Disease: Improving Global Outcomes (KDIGO) defines AKI as an increase in serum creatinine (sCr) ≥ 0.3 mg/dL within 24 h, an increase in sCr ≥ 1.5 times baseline, or a urine volume < 0.5 mL/kg/hr for 6 h [6].

Chronic kidney disease (CKD), which is defined as a glomerular filtration rate (GFR) of < 60 mL/min/1.73^2^, has been demonstrated to be a predictor of AKI following TJA [7, 8]. The GFR is used to measure renal function and to identify renal impairment. However, direct measurement in clinical practice is complex, expensive, and impractical [9]. Instead, estimated GFR (eGFR) is most commonly used to calculate renal function [7]. There are various equations used to calculate eGFR, each factoring in a combination of sCr, age, race, sex, and/or height and weight [10]. The major five equations are the Modification of Diet in Renal Disease (MDRD II) [11] equation, re-expressed MDRD II [12], Cockcroft-Gault (CG) [13], the Mayo Quadratic (Mayo) [14], and the Chronic Kidney Disease Epidemiology Collaboration (CKD-EPI) equations [15]. The differing variables and coefficients used in these equations result in different eGFR values for any specific sCr. As a worsening GFR leads to unfavorable outcomes [16], it is important to identify a consistent calculation that best predicts postoperative AKI.

According to the 2012 KDIGO clinical practice guidelines, the CKD-EPI equation is recommended to calculate eGFR as it has demonstrated the highest accuracy as compared to the other equations [6]. However, a recent study demonstrated the Mayo equation was the most predictive in identifying AKI after cardiovascular surgery [17]. The best equation for predicting postoperative AKI in TJA has not been investigated, and that is the purpose of this study. We sought to evaluate the eGFRs calculated from the five equations, and to identify which equation may be most predictive of postoperative AKI in patients following TJA.

## Methods

### Study population

**Fig. 1:**
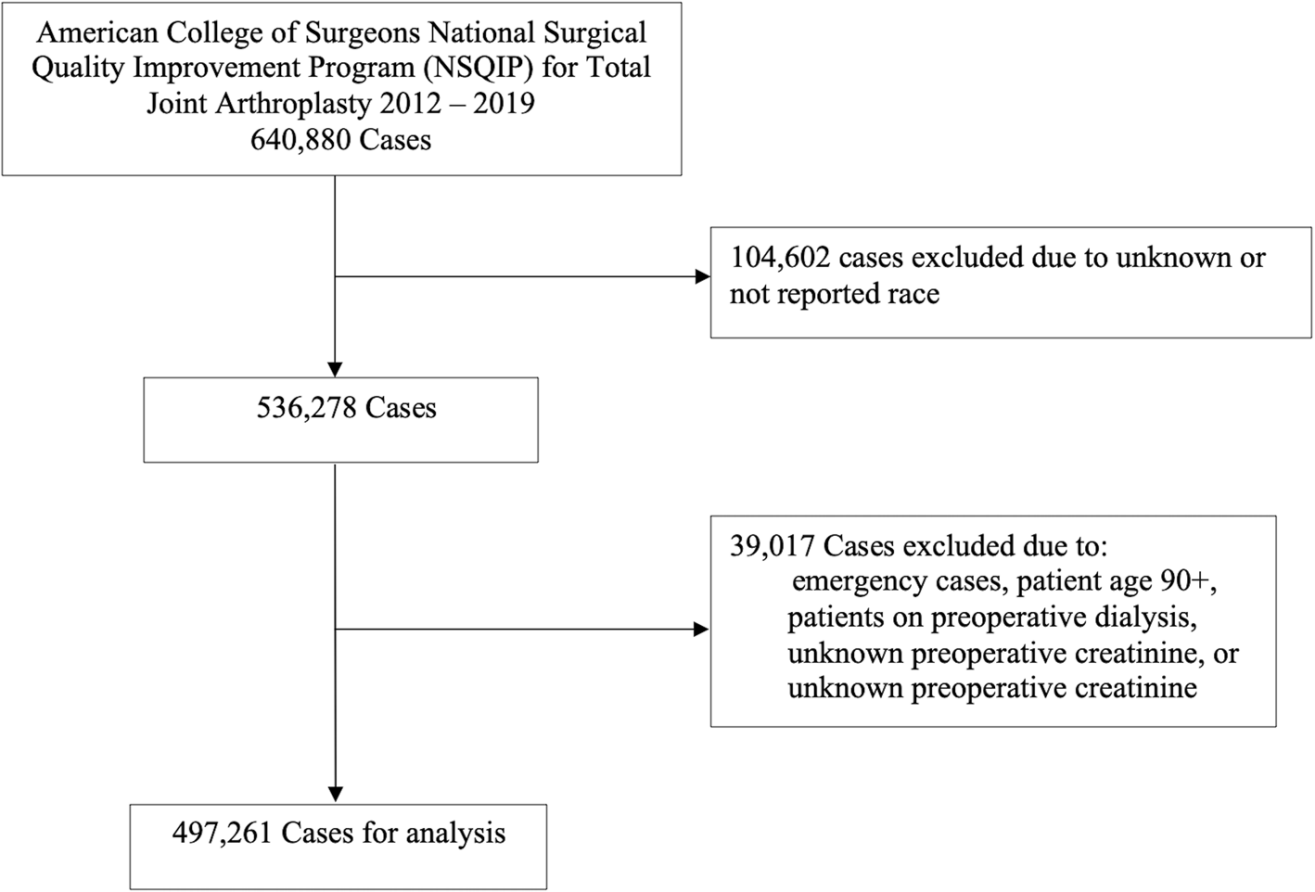
Flow diagram indicating study inclusion and exclusion criteria

A retrospective review was conducted of all 640,880 cases of TJA in the American College of Surgeons National Surgical Quality Improvement Program (ACS-NSQIP) from January 1, 2012 to December 31, 2019. TJA cases were identified with the Current Procedural Terminology (CPT) codes 27,447 (total knee arthroplasty) and 27,130 (total hip arthroplasty). Exclusion criteria included unknown or not reported race, age 90 or older (as NSQIP groups these patients as age 90+), emergency cases, patients on preoperative dialysis, unknown preoperative creatinine, and unknown preoperative height or weight. A total of 143,619 cases were removed due to these criteria, resulting in a total of 497,261 cases included for analysis in this study (Fig. 1).

### Variables

Preoperative factors (age, sex, race, height, weight, medical comorbidities, and preoperative laboratory values), intraoperative factors (surgical duration and procedure type), and complications (progressive renal insufficiency and acute renal failure) were extracted from NSQIP and included in this study. NSQIP collects data for 30 days postoperatively, therefore all complications including AKI are within one month after surgery. Body mass index (BMI) was calculated using height and weight.

The eGFR was calculated using the following equations, utilizing the preoperative sCr taken closest to the time before surgery:

1. MDRD II equation [11]: eGFR = 186 × sCr − 1.154 × Age − 0.203 × (0.742 if female) × (1.210 if African − American)
2. Re-expressed MDRD II equation [12]: eGFR = 175 × sCr − 1.154 × Age − 0.203 × (0.742 if female) × (1.210 if African − American)
3. CG equation [13]: eGFR = [(140 − Age) × Weight/(72 × sCr)] × (0.85 if female)
  This equation is adjusted for body surface area: (1.73 *m*^2^ × CG)/BSA，where BSA = 0.007184 × weight 0.425 × height 0.725
4. Mayo equation [14]: eGFR = exp [1.911 + 5.249/sCr − 2.114/sCr^2^ − 0.00686 × Age − (0.205 if female)], if sCr < 0.8 mg/dL then sCr = 0.8
5. CKD-EPI Equation [15]: eGFR = 141 × min (sCr/κ, 1)α × max (sCr/κ, 1) − 1.209 × 0.993Age × 1.018 [if female] × 1.159 [if African − American], where κ is 0.9 for males and 0.7 for females, α is –0.411 for males and –0.329 for females, min demonstrates the minimum of sCr/κ or 1, and max demonstrates the maximum of sCR/κ or 1 [15].

The preoperative eGFRs calculated by the five different equations were stratified into categories based on KDIGO classification: Stage 1: ≥ 90, Stage 2: < 90–60, Stage 3a: < 60–45, Stage 3b: < 45–30, Stage 4: < 30–15, and Stage 5: < 15 mL/min/1.73 m^2^ [6].

### Statistical analysis

Cases were stratified into two groups based on the development of AKI postoperatively and assessed for differences in preoperative factors and intraoperative factors. Descriptive statistics were reported for continuous variables as mean ± standard deviation and for categorical variables as frequencies and percentages. Univariate analysis for continuous and categorical variables was conducted by using analysis of variance and chi-squared or Fisher's exact test, as appropriate. Multivariate logistic regression models were used to evaluate the odds of developing AKI postoperatively, adjusted for age, sex, BMI, preoperative laboratory values (creatinine, albumin, and hematocrit), patient comorbidities (diabetes, congestive heart failure, chronic obstructive pulmonary disease, hypertension, and smoking status), and surgical duration for each of the five equations. Results of the multivariate regression model were reported as odds ratios (ORs) with 95% confidence intervals (CIs). Akaike information criterion (AIC) was used to compare the fit of each model in predicting AKI postoperatively, and receiver operating curves (ROC) were generated for each Logistic regression model, with the area under the curve (AUC) calculated for each ROC. Due to the large sample size of this study, an alpha value was accepted at 0.01. Statistical analyses were performed by utilizing Stata software, version 17.0 (StataCorp LLC, College Station, TX, USA).

## Results

Of the 497,261 cases included in this study, 777 (0.16%) patients developed AKI. Table 1 shows the baseline and perioperative characteristics of the study population. The mean age of the AKI cohort was 59 ± 14 years old and 349 (45%) were female. The mean BMI of the AKI cohort was 35 ± 8.1 kg/m^2^.

**Table 1:** Patient demographics and perioperative characteristics

Variable	***n*** (%)			***P***-Value
	All Cases: ***n*** = 497,261	AKI: ***n*** = 777	No AKI: ***n*** = 496,484	
**Female Gender**	294,369 (59)	349 (45)	294,369 (59)	<0.001
**Age (year)**	55 ± 13	59 ± 14	55 ± 13	<0.001
**Race**				<0.001
White	435,993 (88)	617 (79)	435,376 (88)	
Black	46,202 (9.3)	148 (19)	46,054 (9.3)	
Asian	10,614 (2.1)	8 (1.0)	10,606 (2.1)	
Native American, Hawaiian, or Pacific Islander	4452 (0.9)	4 (0.5)	4448 (0.0)	
BMI (kg/m^2^)	32 ± 6.7	35 ± 8.1	32 ± 6.7	<0.001
Preoperative Serum Creatinine (mg/dL)	0.9 ± 0.3	1.3 ± 0.7	0.9 ± 0.3	<0.001
Hematocrit (%)^a^	41 ± 4.1	39 ± 5.2	41 ± 4.1	<0.001
Albumin (g/dL)^b^	4.1 ± 0.4	3.9 ± 0.5	4.1 ± 0.4	<0.001
Diabetes Mellitus	80,003 (16)	273 (35)	79,730 (16)	<0.001
Hypertension	313,447 (63)	699 (90)	312,748 (63)	<0.001
Congestive Heart Failure	1753 (0.4)	30 (3.9)	1723 (0.4)	<0.001
Chronic Obstructive Pulmonary Disease	18,998 (3.8)	92 (12)	18,906 (3.8)	<0.001
Smoker	49,643 (10)	94 (12)	49,549 (10)	0.049
**Type of Surgery**				
THA	188,939 (38)	287 (37)	188,652 (38)	0.543
TKA	308,322 (62)	490 (63)	307,832 (62)	
Surgical Duration (min)	93 ± 38	103 ± 46	93 ± 38	<0.001

Data are expressed as number of patients (%) or mean ± standard deviation

^a^Values available for 489,607 total cases

^b^Values available for 301,393 total cases

Regarding preoperative factors, the AKI and non-AKI cohorts differed significantly by sex (*P* < 0.001), age (*P* < 0.001), race (*P* < 0.001), BMI (*P* < 0.001), hematocrit (*P* < 0.001), albumin (*P* < 0.001), diabetes mellitus (*P* < 0.001), hypertension (*P* < 0.001), congestive heart failure (*P* < 0.001), and chronic obstructive pulmonary disease (*P* < 0.001). The two cohorts did not differ significantly by smoking status (*P* = 0.049). Intraoperatively, the two cohorts also differed significantly by surgical duration (*P* < 0.001) (Table 1).

The results of the eGFR for each of the five equations is summarized in Table 2. The equation with the highest calculated mean eGFR was CG equation (98.6 ± 32.7), followed by the Mayo equation (97.3 ± 19.6), CKD-EPI equation (91.6 ± 17.1), MDRD II equation (86.6 ± 26.4), and finally the re-expressed MDRD II equation had the lowest calculated mean eGFR (75.1 ± 28.8).

**Table 2:** Distribution of patients by preoperative eGFR based on each of the five equations

	MDRD II	Re-Expressed MDRD II	CG	Mayo	CKD-EPI
Mean eGFR	86.6 ± 26.4	75.1 ± 28.8	98.6 ± 32.7	97.3 ± 19.6	91.6 ± 17.1
≥90	203,028 (41)	115,455 (23)	286,132 (58)	351,417 (71)	283,794 (57)
≥60, <90	233,171 (47)	226,453 (46)	163,614 (33)	122,585 (25)	191,771 (39)
≥45, <60	45,253 (9.1)	107,606 (22)	34,820 (7.0)	14,332 (2.9)	15,663 (3.2)
≥30, <45	13,206 (2.7)	40,494 (8.1)	10,868 (2.2)	6,404 (1.3)	4,864 (1.0)
≥30, <15	2185 (0.4)	6666 (1.3)	1517 (0.3)	2021 (0.4)	959 (0.2)
≤15	418 (0.1)	587 (0.1)	310 (0.1)	502 (0.1)	210 (0.0)

Data are expressed as the number of patients (%)

MDRD II: Modification of Diet in Renal Disease, CG: Cockcroft-Gault, Mayo: Mayo Clinic Quadratic, CKD-EPI: Chronic Kidney Disease Epidemiology Collaboration

The results of the Logistic regression analysis are outlined in Table 3. Lower preoperative eGFR was significantly associated with an increased risk of developing AKI following TJA. The Mayo equation had the best fit of the equations to predict postoperative AKI (AIC = 6546; AUC = 0.712).

**Table 3:** Logistic regression analysis of odds of developing AKI by each of the five equations

Equation	Acute Kidney Injury Odds Ratio (95%CI)	***P***-Value	AIC	AUC
**MDRD II**	0.78 (0.74–0.82)	<0.001	6599	0.721
**Re-Expressed MDRD II**	0.86 (0.82–0.90)	<0.001	6660	0.658
**CG**	0.78 (0.75–0.82)	<0.001	6588	0.689
**Mayo**	0.74 (0.70–0.78)	<0.001	6546	0.712
**CKD-EPI**	0.75 (0.71–0.80)	<0.001	6628	0.707

MDRD II: Modification of Diet in Renal Disease, CG: Cockcroft-Gault, Mayo: Mayo Clinic Quadratic, CKD-EPI: Chronic Kidney Disease Epidemiology Collaboration

## Discussion

Although there were similarities in eGFR between the five equations, the distribution of patients in the various KDIGO categories varied significantly. The Mayo equation classified more patients in higher eGFR groups, while the re-expressed MDRD II equation classified more patients in lower eGFR groups. For example, the proportion of patients defined as having eGFR < 60 mL/min/1.73m^2^ was 22% when calculated using the re-expressed MDRD II equation, in contrast to just 2.9% of patients when using the Mayo equation. The variability seen among these equations in the presence of various eGFR categories might be ascribed to differences in the variables and study populations used in their calculation. For instance, the CG equation adjusts for body surface area, the MDRD equation has demonstrated better performance in those with impaired kidney function, and the Mayo equation has demonstrated superior performance in those with preserved renal function [11, 13, 14].

The fact that the variability in eGFR calculated in the same patient depends on which equation was used may have profound significance. Patients may be stratified into different KDIGO categories, and therefore may or may not be identified as having a preoperative renal impairment. This may affect subsequent decisions about whether these patients receive perioperative optimization treatments. Since the literature shows worse outcomes in patients who acquire AKI, it is essential to identify the best method of determining which patients are at the highest risk of developing postoperative AKI and who may benefit from preoperative identification and treatment. A more sensitive rather than specific approach may be best in order to capture more patients with AKI and be able to treat them accordingly.

According to KDIGO clinical practice guidelines, the CKD-EPI equation is recommended to calculate eGFR since it has the highest accuracy as compared to the other equations [6]. However, our results demonstrated that the Mayo equation was a better predictor of the development of AKI postoperatively. In a recent study evaluating these five equations in patients undergoing cardiovascular surgery, Jo *et al*. similarly found that the Mayo equation had the greatest accuracy in predicting postoperative AKI than the other four equations [17]. However, orthopedic literature on eGFR equations remains lacking. The literature shows that AKI following TJA is associated with significant morbidity, increased length of stay, and possible additional therapy including hemodialysis [8, 16, 18]. Thus, being able to identify patients at high risk of developing AKI following TJA may positively affect outcomes and reduce costs.

There are several limitations to this study. First, the number of patients who acquired postoperative AKI in our cohort was much less than previous studies. This might be because NSQIPs definition of AKI underestimates the incidence of AKI [19]. Studies utilizing NSQIP may only capture patients with more severe AKI, giving the perception of low incidence but high mortality [20]. Therefore, the incidence of AKI following TJA is likely to be much higher than what was observed in this study. Another limitation is that this study did not assess patient demographic and comorbidity characteristics as independent predictors of AKI. Also, the hospitals participating in this database may not be reflective of the national population as they tend to be more academic and have more resources [21]. However, NSQIP data have been proven highly reliable through the use of internal audits and clinical data collector reviews [22].

## Conclusions

In conclusion, decreasing preoperative eGFR as calculated by each of the five equations was independently associated with an increased risk of AKI following TJA. Although each equation had significant predictive ability, the Mayo equation had the most successful model in predicting AKI in patients undergoing TJA. The results of this study may allow providers to make a more informed decision when identifying patients at risk of postoperative AKI and underscores the need for further investigation and standardization in assessing AKI in arthroplasty surgery.

## Data Availability

The dataset was extracted from a national database and is available upon request.

## Abbreviations

TJA: Total joint arthroplasty
AKI: Acute kidney injury
KDIGO: Kidney Disease: Improving Global Outcomes
sCR: Serum creatinine
CKD: Chronic kidney disease
GFR: Glomerular filtration rate
eGFR: Estimated glomerular filtration rate
MDRD II: Modification of Diet in Renal Disease
CG: Cockcroft-Gault
Mayo: Mayo Quadratic Equation
CKD-EPI: Chronic Kidney Disease Epidemiology Collaboration
ACS-NSQIP: American College of Surgeons National Surgical Quality Improvement Program
CPT: Current Procedural Terminology
BMI: Body mass index
BSA: Body surface area
ORs: Odds ratios
CIs: Confidence intervals
AIC: Akaike information criterion
ROC: Receiver operating curve
AUC: Area under the curve
